# Descriptor Selection via Log-Sum Regularization for the Biological Activities of Chemical Structure

**DOI:** 10.3390/ijms19010030

**Published:** 2017-12-22

**Authors:** Liang-Yong Xia, Yu-Wei Wang, De-Yu Meng, Xiao-Jun Yao, Hua Chai, Yong Liang

**Affiliations:** 1 State Key Laboratory of Quality Research in Chinese Medicines, Macau University of Science and Technology, Macau 999078, China; xia2yin1234@gmail.com (L.-Y.X.); wangyw09@gmail.com (Y.-W.W.); xjyao@must.edu.mo (X.-J.Y.); chch890113@gmail.com (H.C.); 2 Ministry of Education Key Lab of Intelligent Networks and Network Security, Xi’an Jiaotong University, Xi’an 710049, China; dymeng@mail.xjtu.edu.cn

**Keywords:** QSAR, biological activity, descriptor selection, regularization, log-sum

## Abstract

The quantitative structure-activity relationship (QSAR) model searches for a reliable relationship between the chemical structure and biological activities in the field of drug design and discovery. (1) Background: In the study of QSAR, the chemical structures of compounds are encoded by a substantial number of descriptors. Some redundant, noisy and irrelevant descriptors result in a side-effect for the QSAR model. Meanwhile, too many descriptors can result in overfitting or low correlation between chemical structure and biological bioactivity. (2) Methods: We use novel log-sum regularization to select quite a few descriptors that are relevant to biological activities. In addition, a coordinate descent algorithm, which uses novel univariate log-sum thresholding for updating the estimated coefficients, has been developed for the QSAR model. (3) Results: Experimental results on artificial and four QSAR datasets demonstrate that our proposed log-sum method has good performance among state-of-the-art methods. (4) Conclusions: Our proposed multiple linear regression with log-sum penalty is an effective technique for both descriptor selection and prediction of biological activity.

## 1. Introduction

**Figure 1:**
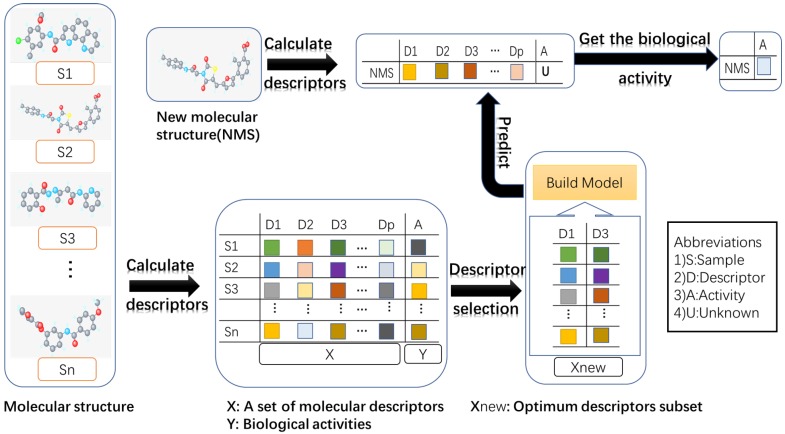
The flow diagram shows the process of QSAR modeling. (1) Collecting molecular structures and their activities; (2) calculating molecular descriptors, which can produce thousands of parameters for each molecular structure; (3) removing redundant or irrelevant descriptors via descriptor selection; (4) building the model with the optimum descriptor subset; (5) predicting the biological activity of a new molecular structure using the established model. Different color blocks represent different values.

The quantitative structure-activity relationship (QSAR) model searches for a reliable relationship between chemical the structure and biological activities in the field of drug design and discovery [1]. In the study of QSAR, the chemical structure is encoded by a substantial number of descriptors, such as thermodynamic, shape descriptors, etc. Generally, only a few descriptors that are relevant to biological activities are of interest to the QSAR model. Descriptor selection aims to eliminate redundant, noisy and irrelevant descriptors [2]. The flow diagram shows the process of QSAR modeling in Figure 1.

Generally, descriptor selection techniques can be categorized into four groups in the study of QSAR: classical methods, artificial intelligence-based methods, miscellaneous methods and regularization methods.

The classical methods have been proposed in the study of QSAR; as an example, forward selection adds the most significant descriptors until none improves the model to a statistically-significant extent. Backward elimination starts with all candidate descriptors, subsequently deleting descriptors without any statistical significance. Generally, stepwise regression builds a model by adding or removing predictor variables based on a series of F-tests or *t*-tests. The variable selection and modeling method based on the prediction [3] uses leave-one-out cross-validation ($Q^{2}$), predicted to select meaningful and important descriptors. Leaps-and-bounds regression [4] selects a subset of descriptors based on the residual sum of squares (RSS).

Recently, artificial intelligence-based methods have been designed for descriptor selection, such as the genetic algorithm [5], which uses the code, selection, exchange and mutation operations to select the important descriptors. Particle swarm optimization [6] has a series of initial random particles and then selects the descriptors by updating the velocity and positions. Artificial neural networks [7] are composed of many artificial neurons that are linked together according to a specific network architecture and select input nodes (descriptors) to predict the output node (biological activity). Simulated annealing [8] can be performed with the Metropolis algorithm based on Monte Carlo techniques, which performs descriptor selection. Frank et al. [9] used Bayesian regularized artificial neural networks with automatic relevance determination (ARD) in the study of QSAR. ARD has the capacity to allow the network to estimate the importance of each input, neglects irrelevant or highly correlated indices in the modeling and uses the most important variables for modeling the activity data. The ant colony system [10], inspired by real ants, searches a path, which is connected to a number of selected descriptors, between the colony and a source of food.

The miscellaneous methods used for descriptor selection in the development of QSAR include *K* nearest neighbor (KNN) [11], the replacement method (RM) [12], the successive projections algorithm (SPA) [13] and uninformative variable elimination-partial least squares (UVE-PLS) [14], just to name a few. KNN uses a similarity measure (Euler distance) to select the descriptor and predict the biological activity. RM has the capacity to find an optimal subset of the descriptors via the standard deviation. SPA is a simple operation to eliminate collinearity to reduce the descriptors. UVE-PLS has been proposed to increase the predictive ability of the standard PLS method via eliminating the variables that cannot contribute to the model and to make a comparison between experimental variables and added noise variables with respect to the degree of contribution to the model.

The regularization is an effective technique in descriptor selection and has been used in QSRR [15], QSPR [16] and QSTR [17] in the field of chemometrics. However, some individuals have poured their interest and attention into the study of QSAR. For example, LASSO ($L_{1}$) (least absolute shrinkage and selection operator) [18] has the capacity to perform descriptor selection. Algamal et al. proposed the $L_{1}$-norm to select the significant and meaningful descriptors for anti-hepatitis C virus activity of thiourea derivatives in the QSAR classification model [19]. Xu et al. proposed $L_{1/2}$ [20] regularization, which has more sparsity. Algamal et al. proposed a penalized linear regression model with the $L_{1/2}$-norm to select the significant and meaningful descriptors [21]. Theoretically, the $L_{0}$ regularization produces better solutions with more sparsity [22], but it is an NP problem. Therefore, Candes et al. proposed the log-sum penalty [23], which approximates the $L_{0}$ regularization much better.

**Figure 2:**
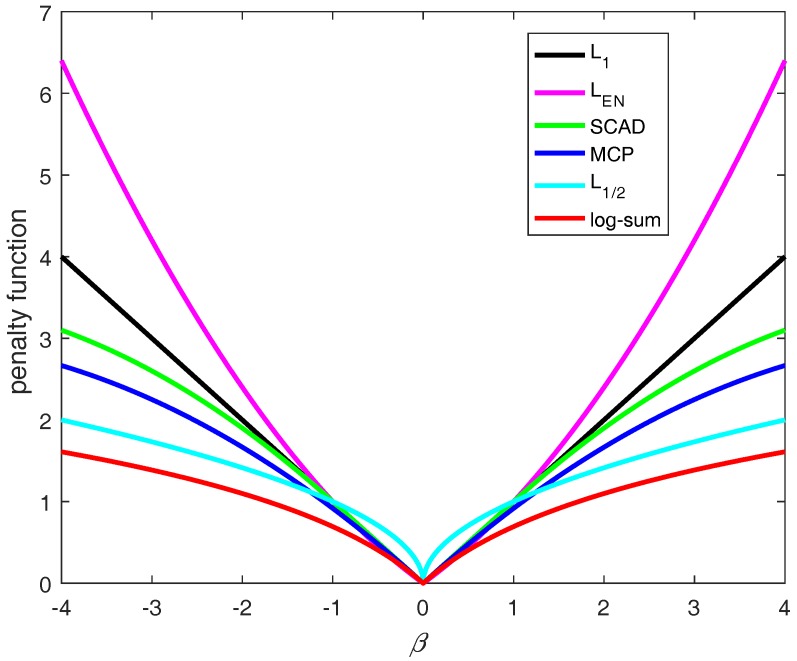
$L_{1}$ and $L_{EN}$ are convex, and SCAD, MCP, $L_{1/2}$ and log-sum are non-convex. The log-sum approximates to $L_{0}$.

In this paper, we utilized the log-sum penalty, which is non-convex in Figure 2. A coordinate descent algorithm, which uses novel univariate log-sum thresholding for updating the estimated coefficients, has been developed for the QSAR model. Experimental results on artificial and four QSAR datasets demonstrate that our proposed log-sum method has good performance among state-of-the-art methods. The structure of this paper is organized as follows: Section 2 introduces a coordinate descent algorithm, which uses novel univariate log-sum thresholding for updating the estimated coefficients and gives a detailed description of the datasets. In Section 3, we discuss the experimental results on simulated data and four QSRA datasets. Finally, we give some conclusions in Section 4.

## 2. Methods

In this paper, there exists a predictor *X* and a response *y*, which represent the chemical structure and corresponding biological activities, respectively. Suppose we have *n* samples, $D=\left(X_{1},y_{1}\right),\left(X_{2},y_{2}\right),\ldots ,\left(X_{n},y_{n}\right)$, where $X_{i}$ = ($x_{i1}$, $x_{i2}$,..., $x_{ip}$) is the *i*-th input pattern with dimensionality *p*, which means $X_{i}$ has *p* descriptors, and $x_{ij}$ denotes the value of descriptor *j* for the *i*-th sample. The multiple linear regression is expressed as:

(1) $$y_{i}=x_{i1}\beta_{1}+\ldots +x_{ip}\beta_{p}+\beta_{0}$$

where $\beta =(\beta_{0},\beta_{1},\ldots ,\beta_{p})$ are the coefficients.

Given *X* and *y*, $\beta_{0},\beta_{1},\ldots ,\beta_{p}$ are estimated based on an objective function. The linear regression of the objective function can be formulated:

(2) $$min\{\frac{1}{2n}∥y-X\beta {∥}^{2}\}$$

where $y={\left(y_{1},......,y_{n}\right)}^{T}$ is the vector of *n* response variables, *X* = {$X_{1}$,$X_{2}$,......,$X_{n}$} is n×p matrix with $X_{i}=\left(x_{i1},\ldots \ldots ,x_{ip}\right)$ and ||.|| denotes the $L_{2}$-norm. When the number of variables is larger than the number of samples (p≫n), this can result in over-fitting. Here, we introduced a penalty function in the objective function to estimate the coefficient. We have rewritten Equation (2):

(3) $$min\{\frac{1}{2n}∥y-X\beta {∥}^{2}+P_{\lambda }\left(\beta \right)\}$$

where $P_{\lambda }\left(\right)$ is a penalty function indexed by the regularized parameter λ>0.

### 2.1. Coordinate Decent Algorithm for Different Thresholding Operators

In this paper, we used the coordinate descent algorithm to implement different penalized multiple linear regression. The algorithm is a “one-at-a-time” algorithm and solves $\beta_{j}$, and other $\beta_{k\neq j}$ (representing the parameters remaining after the *j*-th element is removed) are fixed [22]. Equation (3) can be rewritten as:

(4) $$R\left(\beta \right)=argmin\{\frac{1}{2n}{\left(y_{i}-\left(\sum_{k\neq j}x_{ik}\beta_{k}+x_{ij}\beta_{j}\right)\right)}^{2}+\lambda \sum_{k\neq j}P\left(\beta_{k}\right)+P\left(\beta_{j}\right)\}$$

where *k* represents other variables except the *j*-th variable.

Take the derivative with respect to $\beta_{j}$:

(5) $$\frac{\partial R}{\partial \beta_{j}}=\sum_{i=1}^{n}\left(-x_{ij}\left(y_{j}-\sum_{k\neq j}x_{ik}\beta_{k}-x_{ij}\beta_{j}\right)\right)+\lambda P\left(\beta_{j}\right)=0$$

Denote ${\tilde{y}}_{i}^{\left(j\right)}=\sum_{k\neq j}x_{ik}\beta_{k}$, ${\tilde{r}}_{i}^{\left(j\right)}=y_{i}-{\tilde{y}}_{i}^{\left(j\right)}$, $w_{j}=\sum_{i=1}^{n}x_{ij}{\tilde{r}}_{i}^{\left(j\right)}$, where ${\tilde{r}}_{i}^{\left(j\right)}$ represents the partial residuals with respect to the *j*-th covariate. To take into account the correlation of descriptors, Zhou et al. have proposed elastic net ($L_{EN}$) [24], which emphasizes a grouping effect. The $L_{EN}$ penalty function is given as follows:

(6) $$P\left(\beta \right)=\left(1-a\right)\frac{1}{2}{∥\beta ∥}_{L_{2}}^{2}+a{∥\beta ∥}_{L_{1}}$$

The penalty function of $L_{EN}$ is a combination of the $L_{1}$ penalty (a=1) and the ridge penalty (a=0). Therefore, Equation (5) is rewritten as follows:

(7) $$\frac{\partial R}{\partial \beta_{j}}=\sum_{i=1}^{n}\left(-x_{ij}\left(y_{j}-\sum_{k\neq j}x_{ik}\beta_{k}-x_{ij}\beta_{j}\right)\right)+\lambda \left(1-a\right)\beta_{j}+\lambda a=0$$

Donoho et al. proposed the univariate solution [25] for a $L_{EN}$-penalized regression coefficient as follows:

(8) $$\beta_{j}=f_{L_{EN}}\left(w_{j},\lambda ,a\right)=\frac{S(w_{j},\lambda a)}{1+\lambda (1-a)}$$

where $S(w_{j},\lambda a)$ is the soft thresholding operator for the $L_{1}$ if a is equal to one; Formula (8) can be rewritten as follows:

(9) $$\beta_{j}=Soft\left(w_{j},\lambda \right)=\left\{\begin{array}{cc} w_{j}+\lambda & \mathrm{if}\;w_{j}<-\lambda \\ w_{j}-\lambda & \mathrm{if}\;w_{j}>\lambda \\ 0 & \mathrm{if}\;-\lambda \leq w_{j}\leq \lambda \end{array}\right.$$

Fan et al. have proposed the smoothly clipped absolute deviation (SCAD) [26], which can produce a sparse set of solutions and approximately unbiased coefficients for large coefficients. The penalty function is shown as follows:

(10) $$p_{\lambda ,a}\left(\beta \right)=\left\{\begin{array}{cc} \lambda \beta & \mathrm{if}\;\beta \neq \lambda \\ \frac{a\lambda \beta -\frac{1}{2}\left(\beta^{2}+\lambda^{2}\right)}{a-1} & \mathrm{if}\;\lambda <\beta <a\lambda \\ \frac{\lambda (a^{2}-1)}{2(a-1)} & \mathrm{if}\;\beta >a\lambda \end{array}\right.$$

Additionally, the SCAD thresholding operator is given as follows:

(11) $$\beta_{j}=f_{\mathrm{SCAD}}\left(w_{j},\lambda ,a\right)=\left\{\begin{array}{cc} S(w_{j},\lambda ) & \mathrm{if}\;|w_{j}|<2\lambda \\ \frac{S(w_{j},a\lambda /\left(a-1\right))}{1-1/(a-1)} & \mathrm{if}\;2\lambda <|w_{j}|\leq a\lambda \\ w_{j} & \mathrm{if}\;|w_{j}|>a\lambda \end{array}\right.$$

Similar to the SCAD penalty, Zhang et al. have proposed the maximum concave penalty (MCP) [27]. The formula of the penalty function is shown as:

(12) $$p_{\lambda ,a}\left(\beta \right)=\left\{\begin{array}{cc} \lambda \beta & \mathrm{if}\;\beta \leq \gamma \lambda \\ \frac{1}{2}\gamma \lambda^{2} & \mathrm{if}\;\beta >\gamma \lambda \end{array}\right.$$

Additionally, the MCP thresholding operator is given as follows:

(13) $$\beta_{j}=f_{\mathrm{MCP}}\left(w_{j},\lambda ,\gamma \right)=\left\{\begin{array}{cc} \frac{S(w_{j},\lambda )}{1-1/\gamma } & \mathrm{if}\;|w_{j}|\leq \gamma \lambda \\ w_{j} & \mathrm{if}\;|w_{j}|>\gamma \lambda \end{array}\right.$$

where γ is the experience parameter.

Xu et al. proposed $L_{1/2}$ regularization [20]. Formula (3) can be rewritten:

(14) $$min\{\frac{1}{2n}∥y-X\beta {∥}^{2}+\lambda \sum_{j}^{p}|\beta_{j}{|}^{\frac{1}{2}}\}$$

and the univariate half thresholding operator for a $L_{1/2}$-penalized linear regression coefficient is as follows:

(15) $$\beta_{j}=Half\left(w_{j},\lambda \right)=\left\{\begin{array}{cc} \frac{2}{3}w_{j}\left(1+\cos\frac{2(\pi -\phi_{\lambda }\left(w_{j}\right))}{3}\right) & \mathrm{if}\;|w_{j}|>\frac{3}{4}{\left(\lambda \right)}^{\frac{2}{3}}\\ 0 & otherwise \end{array}\right.$$

where $\phi_{\lambda }\left(w\right)=\frac{\lambda }{8}{\left(\frac{\left|w\right|}{3}\right)}^{-\frac{3}{2}}$.

In this paper, we applied the log-sum penalty to the linear regression model. We could rewrite Formula (3) as follows:

(16) $$min\{\frac{1}{2n}{∥y-X\beta ∥}^{2}+\lambda \sum_{j}^{p}log(|\beta_{j}\left|+\varepsilon )\right\}$$

where ε>0 should be set arbitrarily small, to make the log-sum penalty closely resemble the $L_{0}$-norm. Equation (16) has a local minimal. The proof is given in the Appendix A:

(17) $$\beta_{j}=f_{log-sum}\left(w_{j},\lambda ,\varepsilon \right)=D\left(w_{j},\lambda ,\varepsilon \right)=\left\{\begin{array}{cc} sign\left(w_{j}\right)\frac{c_{1}+{\sqrt{c}}_{2}}{2} & \mathrm{if}\;c_{2}>0\\ 0 & \mathrm{if}\;c_{2}\leq 0 \end{array}\right.$$

where $\lambda >0,0<\varepsilon <\sqrt{\lambda },c_{1}=\omega_{j}-\varepsilon$ and $c_{2}=c_{1}^{2}-4\left(\lambda -w_{j}\varepsilon \right)$.

**Figure 3:**
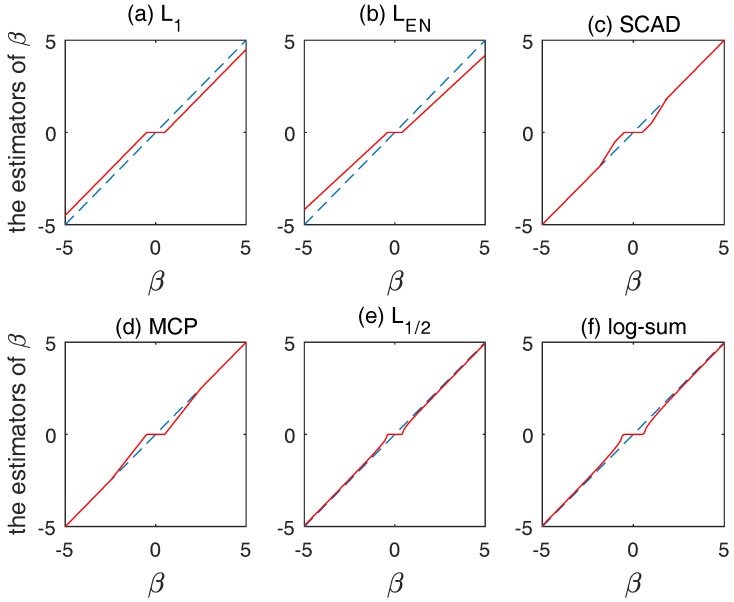
Plot of thresholding functions for: (**a**) $L_{1}$; (**b**) $L_{EN}$; (**c**) SCAD; (**d**) MCP; (**e**) $L_{1/2}$; and (**f**) log-sum.

According to different thresholding operators, we can define three properties for to satisfy the coefficient estimator, unbiasedness, sparsity and continuity, in Figure 3.

### 2.2. Dataset

#### 2.2.1. Simulated Data

In this work, we constructed the simulation. The process of the construction was given as follows:

Step I: The simulated dataset was generated from multiple linear regression using the normal distribution to produce X. Here, the number of row is sample *n* and the number of column is variable *p*.

(18) $$y=X\beta +\overset{intercept}{\overset{︷}{\sigma \epsilon }},\epsilon ∼N\left(0,1\right)$$

where $y={\left(y_{1},\ldots ,y_{n}\right)}^{T}$ is the vector of *n* response variables, X = {$X_{1}$, $X_{2}$, ..., $X_{n}$} is the generated matrix with $X_{i}=\left(x_{i1},\ldots ,x_{ip}\right)$, $\epsilon ={\left(\epsilon_{1},\ldots ,\epsilon_{n}\right)}^{T}$ is the random error and σ controls the signal to noise.

Step II: Add a different correlation parameter ρ to the simulation data.

(19) $$x_{ij}=\rho \times x_{11}+\left(1-\rho \right)x_{ij},i∼\left(1,\ldots ,n\right),j∼\left(2,3,4,5,6\right)$$

Step III: In order to get a high quality model and variable selection, the coefficients (20) are set in advance from 1–20.

(20) $$\beta =\overset{2000}{\overset{︷}{\underset{20}{\underset{︸}{2,-2,-1,1.5,3,2.5,3,2,\ldots ,2,}}\underset{1980}{\underset{︸}{0,0,0,\ldots ,0}}}}$$

where β is the coefficient.

Step IV: We can get y from Equations (18)–(20).

In the simulation study, we firstly generated 100 groups of data with different sample sizes n=100 and n=200. Secondly, the correlation coefficient ρ=0.2,0.4 and the noise control parameter σ=0.3,0.9, were considered in the model. Thirdly, the coefficients (20) are set in advance. Fourthly, the multiple linear regression with different penalties to select variables and build the model, including our proposed method, was used. Finally, due to the generation of 100 groups of data, the results obtained by different methods need to be averaged.

#### 2.2.2. Real Data

We could obtain four public QSAR datasets, including the global half-life index [28], endocrine disruptor chemical (EDC) estrogen receptor (ER)-binding [29], (Benzo-)Triazoles toxicity in Daphnia magna [30] and apoptosis regulator Bcl-2 [31]. A brief description of these datasets is shown in Table 1. We utilized random sampling to divide datasets into training datasets and test datasets (80% for the training set and 20% for the test set [32]). Six commonly-used parameters in regression problems are employed to evaluate the model performance, including the square correlation coefficients of the leave-one-out cross-validation ($Q_{LOO}^{2}$), the root mean squared error of cross-validation ($RMSE_{CV}$), the square correlation coefficients of fitting for the training set ($R_{train}^{2}$), the root mean squared error for the training set ($RMSE_{train}$), the square correlation coefficients of fitting for the test set ($R_{test}^{2}$) and the root mean squared error for the test set ($RMSE_{test}$). According to existing literature [33], we have learned that the value of $Q_{LOO}^{2}$ is not the best measure for QSAR model evaluation. Therefore, we poured more interest and attention into ($R_{test}^{2}$) and ($RMSE_{test}$).
**Algorithm:** A coordinate descent algorithm for log-sum penalized multiple linear regression.*Step 1*: Initialize all $\beta_{j}\left(m\right)=0\left(j=1,2,3,\ldots ,p\right),\lambda ,\varepsilon$,set m=0;*Step 2*: Calculate the function (16) based on β(m)*Step 3*: Update each $\beta_{j}\left(m\right)$ and cycle j=1,2,3,…,p              Step 3.1: ${\tilde{r}}_{i}^{\left(j\right)}\left(m\right)=y_{i}\left(m\right)-{\tilde{y}}_{i}^{\left(j\right)}\left(m\right)=y_{i}\left(m\right)-\sum_{k\neq j}x_{ik}\beta_{k}\left(m\right)$                            and $w_{j}\left(m\right)=x_{ij}\left(r_{i}\left(m\right)-{\tilde{r}}_{i}^{\left(j\right)}\left(m\right)\right)$              Step 3.2: Update $\beta_{j}\left(m\right)=D\left(w_{j},\lambda ,\varepsilon \right)$*Step 4*: Let m←(m+1),β(m+1)←β(m)*Step 5*: Repeat Steps 2 and 3 until β(m) converges

## 3. Results

In this work, five methods are compared to our proposed method, including multiple linear regression with $L_{EN}$, $L_{1}$, SCAD, MCP and $L_{1/2}$ penalties, respectively.

### 3.1. Analyses of Simulated Data

Table 2 and Table 3 describe the number of variables that are selected (non-zero coefficient) by different methods within 2000 variables and within pre-set variables (20), respectively. For example, when n=200,ρ=0.4 and σ=0.9, the average number of variables selected is 23.73 within 2000 variables by the log-sum in Table 2. In pre-set variables (20), we got 19.95 variables by the log-sum in Table 3. Therefore, we could calculate the average accuracy (19.95÷23.73×100%=84.07%) for the simulation datasets obtained by log-sum in Table 4. From Table 2, Table 3 and Table 4, for example, when the correlation parameter ρ and the noise control parameter σ decrease, the average accuracy of log-sum improves. When n=100 and σ=0.9, the average accuracy of log-sum is from 83.77–98.7%, where the correlation parameter ρ is from 0.4–0.2. When n=200 and ρ=0.4, the results obtained by log-sum are 84.07% and 86.39% with the noise control parameter σ=0.9, 0.3. In addition, compared to other methods, the average accuracy obtained by our proposed log-sum method is better, for example when n=200, ρ=0.4 and σ=0.9, the result of the log-sum is 84.07% higher than 3.19%, 20.20%, 49.20%, 83.22% and 81.74% of the $L_{EN}$, $L_{1}$, SCAD, MCP and $L_{1/2}$. In other words, our proposed log-sum method has the capacity to obtain good performance in the simulation dataset.

### 3.2. Analyses of Real Data

**Figure 4:**
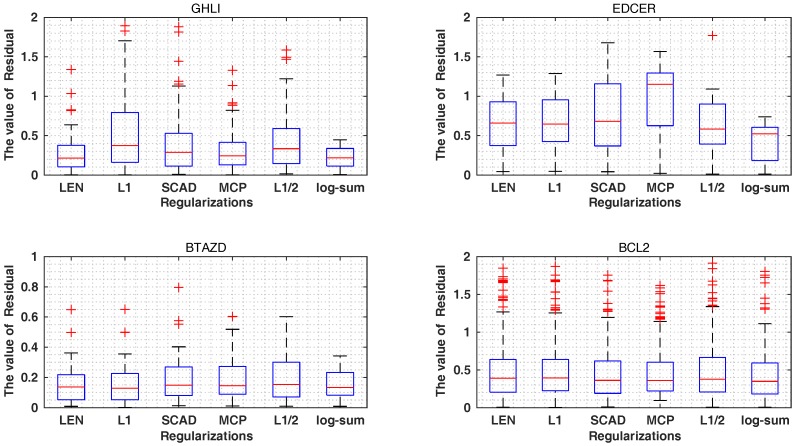
The value of residual ($|y-y^{pred}|$) on different datasets.

**Figure 5:**
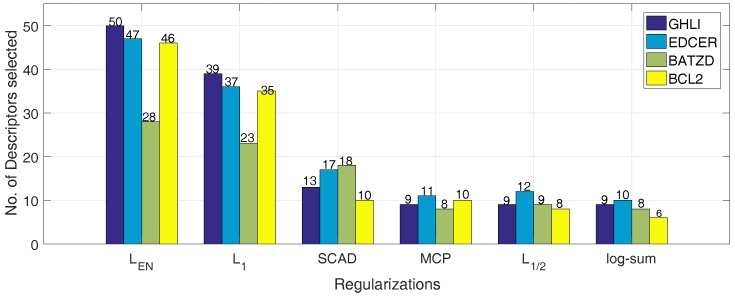
The number of descriptors obtained by the multiple linear regression with the different penalties on different datasets(different colors represent different datasets).

As shown in Table 5 and Figure 4 and Figure 5, the $R_{train}^{2}$ and $RMSE_{train}$ of the $L_{1}$, $L_{1/2}$ and MCP are 0.87, 0.87, 0.88 and 0.64, 0.62, 0.27, better than the values of 0.85, 0.86, 0.88 and 0.69, 0.63, 0.28 of the log-sum for the GHLI, EDCER and BATZD datasets, respectively. However, our proposed log-sum method is the best in terms of $Q^{2}$ and $RMSE_{CV}$. In the BATZD dataset, the $RMSE_{CV}$ obtained by log-sum is 0.23, lower than the values of 0.30, 0.30, 0.30, 0.28 and 0.26 of other methods. In the BCL2 dataset, the $Q^{2}$ obtained by log-sum is 0.75, higher than the 0.51, 0.57, 0.73, 0.73 and 0.67 of other methods. Moreover, a small subset of descriptors was selected by our proposed method; for example, for the EDCER dataset, the result of log-sum is 10, lower than the 47, 36, 17, 11 and 12 of $L_{EN}$, $L_{1}$, SCAD, MCP and $L_{1/2}$. Furthermore, for $R_{Test}^{2}$ and $RMSE_{test}$, for the GHLI dataset, the best method is log-sum (0.75 and 0.88); $L_{EN}$ and $L_{1}$ are second (0.74 and 0.90); MCP is third (0.73 and 0.91); $L_{1/2}$ is fourth (0.72 and 0.92); and the last is SCAD (0.72 and 0.93). Therefore, our proposed method is better than the other methods. In addition, we gave the experimental and predicted values for the four datasets.

First of all, in Table 6, Table 7, Table 8 and Table 9, the number of top-ranked informative descriptors identified by $L_{EN}$, $L_{1}$, SCAD, MCP, $L_{1/2}$ and log-sum is 9, 10, 8 and 6 based on the value of the coefficients. Secondly, the common descriptors are emphasized in bold. Thirdly, as shown in Table 10, the number of descriptors is from the class of 2D. Then, the majority of descriptors are belong to the atom-type electrotopological state and autocorrelation of descriptors types. Finally, the name of the descriptors obtained by the log-sum method is exhibited in Table 11.

## 4. Conclusions

In the field of drug design and discovery, only a few descriptors are of interest to the QSAR model. Therefore, descriptor selection plays an important role in the study of QSAR. In this paper, we proposed univariate log-sum thresholding for updating the estimated coefficients and developed a coordinate descent algorithm for log-sum penalized multiple linear regression.

Both experimental results on artificial and four QSAR datasets demonstrate that our proposed multiple linear regression with log-sum penalty is still better than $L_{1}$, $L_{EN}$, SCAD, MCP and $L_{1/2}$. Therefore, our proposed log-sum method is the effective technique in both descriptor selection and prediction of biological activity.

In this paper, we introduced random sampling, which is easy to use, for QSAR data preprocessing. However, this method does not take into account additional knowledge. Therefore, we plan to integrate a self-paced learning mechanism, which learns easy samples first and then gradually takes into consideration complex samples, making the model more and more mature, with our proposed method in future work.

## Figures and Tables

**Table 1:** A brief description of four public datasets used in the experiments.

Dataset Name	No. of Samples	No. of Descriptors	No. of Samples (Training)	No. of Samples (Test)
**BTAZD**	97	1083	78	19
**EDCER**	129	1089	104	25
**GHLI**	250	1120	200	50
**BCL2**	508	1562	407	101

**Table 2:** The average number of variables selected in total by $L_{EN}$, $L_{1}$, SCAD, MCP, $L_{1/2}$ and log-sum. In bold, the best performance is shown.

	Sample Size	$L_{\mathrm{EN}}$	$L_{1}$	SCAD	MCP	$L_{1/2}$	Log-Sum
ρ=0.2, σ=0.3	n=100	381.60	92.92	19.09	23.36	19.13	**19.00**
	n=200	498.81	34.18	19.03	**19.00**	19.09	**19.00**
ρ=0.2, σ=0.9	n=100	382.24	93.26	27.74	25.79	21.77	**21.54**
	n=200	499.49	95.83	36.48	23.65	23.83	**23.15**
ρ=0.4, σ=0.3	n=100	378.96	93.98	19.26	24.67	19.98	**19.11**
	n=200	495.66	97.51	40.87	24.04	24.42	**23.79**
ρ=0.4, σ=0.9	n=100	379.35	93.46	29.22	26.08	22.48	**22.04**
	n=200	495.64	98.97	40.61	23.95	24.43	**23.73**

**Table 3:** The average number of variables selected with a pre-set value (20) obtained by $L_{EN}$, $L_{1}$, SCAD, MCP, $L_{1/2}$ and log-sum.

	Sample Size	$L_{\mathrm{EN}}$	$L_{1}$	SCAD	MCP	$L_{1/2}$	Log-Sum
ρ=0.2, σ=0.3	n=100	12.23	14.45	19.09	18.81	19.13	19.00
	n=200	16.22	20.00	19.03	19.00	19.09	19.00
ρ=0.2, σ=0.9	n=100	12.24	14.30	19.93	19.42	19.74	19.81
	n=200	16.26	20.00	20.00	20.00	20.00	20.00
ρ=0.4, σ=0.3	n=100	11.84	13.57	18.88	18.40	18.65	18.88
	n=200	15.79	19.99	19.97	19.93	19.96	19.93
ρ=0.4, σ=0.9	n=100	11.88	13.55	19.48	18.81	19.14	19.00
	n=200	15.80	19.99	19.98	19.93	19.97	19.95

**Table 4:** The average accuracy (%) for the simulation data sets obtained by $L_{EN}$, $L_{1}$, SCAD, MCP, $L_{1/2}$ and log-sum. In bold, the best performance is shown.

	Sample Size	$L_{\mathrm{EN}}$	$L_{1}$	SCAD	MCP	$L_{1/2}$	Log-Sum
ρ=0.2, σ=0.3	n=100	3.20%	15.55%	**100.00%**	80.52%	**100.00%**	**100.00%**
	n=200	3.25%	58.51%	**100.00%**	**100.00%**	**100.00%**	**100.00%**
ρ=0.2, σ=0.9	n=100	3.12%	14.44%	98.03%	74.58%	93.34%	**98.80%**
	n=200	3.19%	20.50%	48.86%	82.90%	81.74%	**83.77%**
ρ=0.4, σ=0.3	n=100	3.20%	15.33%	71.85%	75.30%	90.68%	**91.97%**
	n=200	3.26%	20.87%	54.87%	84.57%	83.93%	**86.39%**
ρ=0.4, σ=0.9	n=100	3.19%	20.50%	48.86%	82.90%	81.74%	**83.77%**
	n=200	3.19%	20.20%	49.20%	83.22%	81.74%	**84.07%**

**Table 5:** Experimental results on the four datasets (the results are emphasized by our proposed method in bold and italic).

Datasets	Methods	$R_{\mathrm{train}}^{2}$	${\mathrm{RMSE}}_{\mathrm{train}}$	$Q_{\mathrm{LOO}}^{2}$	${\mathrm{RMSE}}_{\mathrm{cv}}$	$R_{\mathrm{test}}^{2}$	${\mathrm{RMSE}}_{\mathrm{test}}$
***GHLI***	$L_{EN}$	0.87	0.65	0.74	0.68	0.74	0.90
	$L_{1}$	0.87	0.64	0.75	0.67	0.74	0.90
	SCAD	0.84	0.71	0.82	0.62	0.72	0.93
	MCP	0.85	0.68	0.80	0.65	0.73	0.91
	$L_{1/2}$	0.82	0.75	0.81	0.62	0.72	0.92
	***log-sum***	***0.85***	***0.69***	***0.84***	***0.57***	***0.75***	***0.88***
***EDCER***	$L_{EN}$	0.81	0.74	0.70	0.70	0.64	1.23
	$L_{1}$	0.82	0.73	0.73	0.68	0.63	1.25
	SCAD	0.86	0.63	0.74	0.69	0.70	1.12
	MCP	0.83	0.70	0.74	0.69	0.65	1.21
	$L_{1/2}$	0.87	0.62	0.75	0.65	0.64	1.24
	***log-sum***	***0.86***	***0.63***	***0.79***	***0.62***	***0.70***	***1.12***
***BATZD***	$L_{EN}$	0.87	0.28	0.73	0.30	0.60	0.52
	$L_{1}$	0.88	0.28	0.74	0.30	0.60	0.52
	SCAD	0.86	0.30	0.77	0.30	0.62	0.51
	MCP	0.88	0.27	0.83	0.29	0.64	0.50
	$L_{1/2}$	0.86	0.29	0.84	0.26	0.64	0.50
	***log-sum***	***0.88***	***0.28***	***0.88***	***0.23***	***0.68***	***0.47***
***BCL2***	$L_{EN}$	0.75	0.57	0.51	0.53	0.61	0.67
	$L_{1}$	0.74	0.58	0.58	0.51	0.61	0.67
	SCAD	0.72	0.59	0.73	0.45	0.59	0.69
	MCP	0.74	0.57	0.73	0.46	0.58	0.70
	$L_{1/2}$	0.73	0.60	0.68	0.48	0.57	0.70
	***log-sum***	***0.68***	***0.64***	***0.75***	***0.43***	***0.65***	***0.63***

**Table 6:** The 9 top-ranked descriptors identified by $L_{EN}$, $L_{1}$, SCAD, MCP, $L_{1/2}$ and log-sum from the GHLI dataset (the common descriptors are emphasized in bold).

Rank	GHLI					
	$L_{\mathrm{EN}}$	$L_{1}$	SCAD	MCP	$L_{1/2}$	Log-Sum
**1**	***JGI7***	***JGI7***	Mp	***JGI7***	minsCl	***ATSC4c***
**2**	***ETA_Eta_B_RC***	***ETA_Eta_B_RC***	MDEC-44	***ATSC4c***	ATSC1e	***GATS1e***
**3**	***BCUTc-1l***	***BCUTc-1l***	***GATS1e***	***GATS1e***	minaaN	***ATSC1p***
**4**	***Mv***	***Mv***	***ATSC1p***	AATS0e	WPOL	***MATS8m***
**5**	***ATSC4c***	***MDEN-23***	GGI9	meanI	***nHdsCH***	***maxwHBa***
**6**	***MDEN-23***	***ATSC4c***	***maxHBa***	***nHdsCH***	ALogP	***maxHBa***
**7**	***GATS1e***	***GATS1e***	***maxwHBa***	***maxHBa***	nFG12Ring	***ATSC7s***
**8**	ETA_Epsilon_3	***ETA_Epsilon_4***	***MATS8m***	***ATSC7s***	AATS6i	AATS0v
**9**	***ETA_Epsilon_4***	minHCsatu	SIC1	ATS4v	AATSC8m	ATS4p

**Table 7:** The 10 top-ranked descriptors identified by $L_{EN}$, $L_{1}$, SCAD, MCP, $L_{1/2}$ and log-sum from the EDCER dataset (the common descriptors are emphasized in bold).

Rank	EDCER					
	$L_{\mathrm{EN}}$	$L_{1}$	SCAD	MCP	$L_{1/2}$	Log-Sum
**1**	***JGI10***	***JGI10***	***JGI10***	***JGI10***	***JGI10***	***JGI10***
**2**	***VE2_Dt***	***VE2_Dt***	MATS1i	***JGI6***	GATS1c	MATS1c
**3**	***JGI7***	***JGI6***	***AATSC2s***	***AATSC2s***	***GATS2s***	***hmax***
**4**	***AATSC8p***	***AATSC8p***	***hmax***	***AATSC8p***	***hmax***	***nssO***
**5**	***JGI6***	***JGI7***	***JGI6***	***hmax***	***GATS5v***	piPC6
**6**	***hmax***	***hmax***	nBase	nHBint2	nTG12Ring	***nFG12HeteroRing***
**7**	***SpMin4_Bhm***	***SpMin4_Bhm***	GATS8p	nHBd	***nssO***	***maxaaCH***
**8**	***GATS5v***	***GATS5v***	***nFG12HeteroRing***	***maxaaCH***	***maxaaCH***	SHBint2
**9**	***GATS2s***	***GATS2s***	MATS5v	C3SP2	ETA_Beta_ns_d	TIC1
**10**	SpMin5_Bhs	nAcid	***maxaaCH***	SHBint8	MDEC-24	AATSC8m

**Table 8:** The 8 top-ranked descriptors identified by $L_{EN}$, $L_{1}$, SCAD, MCP, $L_{1/2}$ and log-sum from the BATZD dataset (the common descriptors are emphasized in bold).

Rank	BATZD					
	$L_{\mathrm{EN}}$	$L_{1}$	SCAD	MCP	$L_{1/2}$	Log-Sum
**1**	***JGI4***	***JGI4***	***VE2_Dze***	***SpMax1_Bhi***	***SpMax1_Bhi***	***SpMax1_Bhi***
**2**	***VE2_Dze***	***VE2_Dze***	JGI3	MATS5m	GATS1p	GATS1v
**3**	***MATS5v***	***ndS***	***ndS***	***GATS3s***	***ndS***	***GATS3s***
**4**	SdS	***MATS5v***	***CrippenLogP***	C4SP3	GATS3m	GATS8c
**5**	***CrippenLogP***	***CrippenLogP***	***nHother***	***CrippenLogP***	***GATS3s***	naaS
**6**	mindS	***MDEO-22***	minddssS	ALogP	***LipoaffinityIndex***	AATSC4i
**7**	***MDEO-22***	***nF9Ring***	GATS4m	***nHother***	nHsOH	***LipoaffinityIndex***
**8**	maxdS	ETA_Epsilon_4	***nF9Ring***	***ATSC8i***	***ATSC8i***	SpDiam_Dzp

**Table 9:** The 6 top-ranked descriptors identified by $L_{EN}$, $L_{1}$, SCAD, MCP, $L_{1/2}$ and log-sum from the BCL2 dataset (the common descriptors are emphasized in bold).

Rank	BCL2					
	$L_{\mathrm{EN}}$	$L_{1}$	SCAD	MCP	$L_{1/2}$	Log-Sum
**1**	***JGI7***	***AATSC8p***	AATSC4s	***JGI7***	***MATS4s***	***AATSC8p***
**2**	VE2_D	***MATS4s***	***IC2***	***MATS4s***	***IC2***	***IC2***
**3**	***AATSC8p***	***MATS5m***	***MDEN-13***	***IC2***	***E3m***	GATS4s
**4**	***MATS5m***	***IC2***	minHsNH2	***E3m***	***MDEN-13***	***maxHBint2***
**5**	***MATS4s***	***MDEN-13***	***maxHBint2***	GATS8p	***maxHBint2***	***minsOH***
**6**	***IC2***	SpMax1_Bhi	nT8Ring	***MDEN-13***	***minsOH***	SwHBa

**Table 10:** The detailed information of the descriptors obtained by the log-sum method.

Descriptor Type	Class	Descriptor
Autocorrelation	2D	AATS0v; AATSC4i; AATSC8m; ATS4p; ATSC1p;
		ATSC4c; ATSC7s; GATS1e; GATS1v; GATS3s;
		GATS8c; MATS1c; MATS8m; AATSC8p; GATS4s
Atom-type electrotopological state	2D	Hmax; LipoaffinityIndex; maxaaCH; maxHBa; maxwHBa;
		naaS; nssO; SHBint2; maxHBint2; minsOH; SwHBa
Barysz matrix	2D	SpDiam_Dzp
Burden modified eigenvalues	2D	SpMax1_Bhi
Information content	2D	TIC1
Path counts	2D	piPC6
Ring count	2D	nFG12HeteroRing
Topological charge	2D	JGI10
Information content	2D	IC2

**Table 11:** The name of the descriptors obtained by the log-sum method.

Descriptor	Name
AATS0v	Average Broto–Moreau autocorrelation-lag 0/weighted by van der Waals volumes
AATSC4i	Average centered Broto–Moreau autocorrelation-lag 4/weighted by first ionization potential
AATSC8m	Average centered Broto–Moreau autocorrelation-lag 8/weighted by mass
ATS4p	Average centered Broto–Moreau autocorrelation-lag 1/weighted by polarizabilities
ATSC1p	Centered Broto–Moreau autocorrelation-lag 1/weighted by polarizabilities
ATSC4c	Average centered Broto–Moreau autocorrelation-lag 4/weighted by charges
ATSC7s	Average centered Broto–Moreau autocorrelation-lag 7/weighted by I-state
GATS1e	Geary autocorrelation-lag 1/weighted by Sanderson electronegativities
GATS1v	Geary autocorrelation-lag 1/weighted by van der Waals volumes
GATS3s	Geary autocorrelation-lag 3/weighted by I-state
GATS8c	Geary autocorrelation-lag 8/weighted by charges
hmax	Maximum H E-state
JGI10	Mean topological charge index of order 10
LipoaffinityIndex	Lipoaffinity index
MATS1c	Moran autocorrelation-lag 1/weighted by charges
MATS8m	Moran autocorrelation-lag 8/weighted by mass
maxaaCH	Maximum atom-type E-state: :CH:
maxHBa	Maximum E-states for (strong) hydrogen bond acceptors
maxwHBa	Maximum E-states for weak hydrogen bond acceptors
naaS	Count of atom-type E-state::C:-
nFG12HeteroRing	Number of >12-membered fused rings containing heteroatoms (N, O, P, S or halogens)
nssO	Count of atom-type E-state: -O-
piPC6	Conventional bond order ID number of order 6 (ln(1 + x)
SHBint2	Sum of E-state descriptors of strength for potential hydrogen bonds of path length 2
SpDiam_Dzp	Spectral diameter from Barysz matrix/weighted by polarizabilities
SpMax1_Bhi	Largest absolute eigenvalue of Burden-modified matrix - n 1/weighted by the relative first ionization potential
TIC1	Total information content index (neighborhood symmetry of 1-order)
SwHBa	Sum of E-states for weak hydrogen bond acceptors
AATSC8p	Average centered Broto–Moreau autocorrelation-lag 8/weighted by polarizabilities
IC2	Information content index (neighborhood symmetry of 2-order)
GATS4s	Geary autocorrelation-lag 4/weighted by I-state
maxHBint2	Maximum E-State descriptors of strength for potential Hydrogen Bonds of path length 2
minsOH	Minimum atom-type E-state: -OH

## Abbreviations

QSAR	Quantitative structure-activity relationship
QSRR	Quantitative structure-(chromatographic) retention relationships
QSPR	Quantitative structure-property relationship
QSTR	Quantitative structure-toxicity relationship
MLR	Multiple linear regression
MCP	Maximum concave penalty
SCAD	Smoothly clipped absolute deviation
$L_{1}$	LASSO
BTAZD	(Benzo-)Triazoles toxicity in Daphnia magna
EDCER	EDC estrogen receptor binding
GHLI	Global half-life index
BCL2	Apoptosis regulator Bcl-2

## Appendix A. Proof

We first consider the situation $\beta_{j}>0$:

(A1) $$\frac{\partial R}{\partial \beta_{j}}=\sum_{i=1}^{n}\left(-x_{ij}\left(y_{i}-\sum_{k\neq j}x_{ij}\beta_{k}-x_{ij}\beta_{j}\right)\right)+\lambda \frac{1}{\beta_{j}+\varepsilon }=0$$

Based on Equation (A1), the gradient of the log-sum regularization at $\beta_{j}$ can be expressed as:

(A2) $$\frac{\partial R}{\partial \beta_{j}}=\beta_{j}-\omega_{j}+\lambda \frac{1}{\beta_{j}+\varepsilon }=0$$

Denote ${\tilde{y}}_{i}^{\left(j\right)}=\sum_{k\neq j}x_{ik}\beta_{k}$, ${\tilde{r}}_{i}^{\left(j\right)}=y_{i}-{\tilde{y}}_{i}^{\left(j\right)}$, $w_{j}=\sum_{i=1}^{n}x_{ij}{\tilde{r}}_{i}^{\left(j\right)}$, which is equivalent to:

(A3) $$\beta_{j}^{2}-\left(\omega_{j}-\varepsilon \right)\beta_{j}+\left(\lambda -\omega_{j}\varepsilon \right)=0$$

(A4) $$\beta_{j}=\frac{\omega_{j}-\varepsilon \pm \sqrt{{\left(\omega_{j}-\varepsilon \right)}^{2}-4\left(\lambda -\omega_{j}\varepsilon \right)}}{2}$$

let: $c_{1}=\omega_{j}-\varepsilon$,$c_{2}=c_{1}^{2}-4\left(\lambda -\omega_{j}\varepsilon \right)$ Thus, we have:

(1) if $c_{2}<0$, Equation (A3) has no real solution.
(2) if $c_{2}=0$, Equation (A3) has the solution $\beta_{j}=\frac{c_{1}}{2}$.
(3) if $c_{2}>0$, Equation (A3) has the two solutions $\beta_{j1}=\frac{c_{1}-{\sqrt{c}}_{2}}{2}$ and $\beta_{j2}=\frac{c_{1}+{\sqrt{c}}_{2}}{2}$:
  $$\begin{array}{cc} c_{2} & ={\left(\omega_{j}-\varepsilon \right)}^{2}-4\left(\lambda -\omega_{j}\varepsilon \right)\\ & =\omega_{j}^{2}-2\omega_{j}\varepsilon +\varepsilon^{2}-4\lambda +4\omega_{j}\varepsilon \\ & ={\left(\omega_{j}+\varepsilon \right)}^{2}-4\lambda >0\\ & \omega_{j}+\varepsilon >2\sqrt{\lambda }\\ & \omega_{j}-\varepsilon >2\sqrt{\lambda }-2\varepsilon \\ & c_{1}>0 \end{array}$$

Thus, $\beta_{j2}>\beta_{j1}>0$, and it is then easy to obtain that $f^{\prime }\left(\beta_{j}\right)>0$ when $0<\beta_{j}<\beta_{j1}$ or $\beta_{j2}>\beta_{j}$ and $f^{\prime }\left(\beta_{j}\right)<0$ when $\beta_{j1}<\beta_{j}<\beta_{j2}$. Therefore, Equation (16) has a local minimum. For $\beta_{j}<0$, we can prove it in a similar way.
